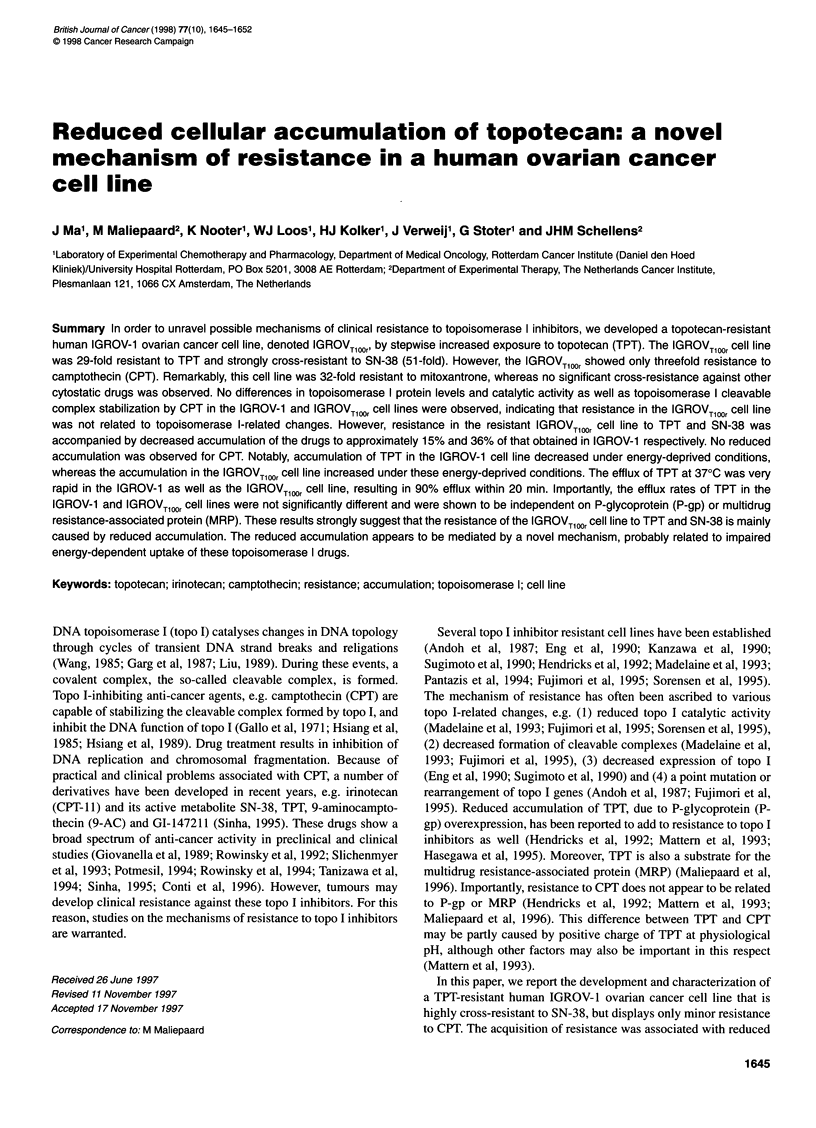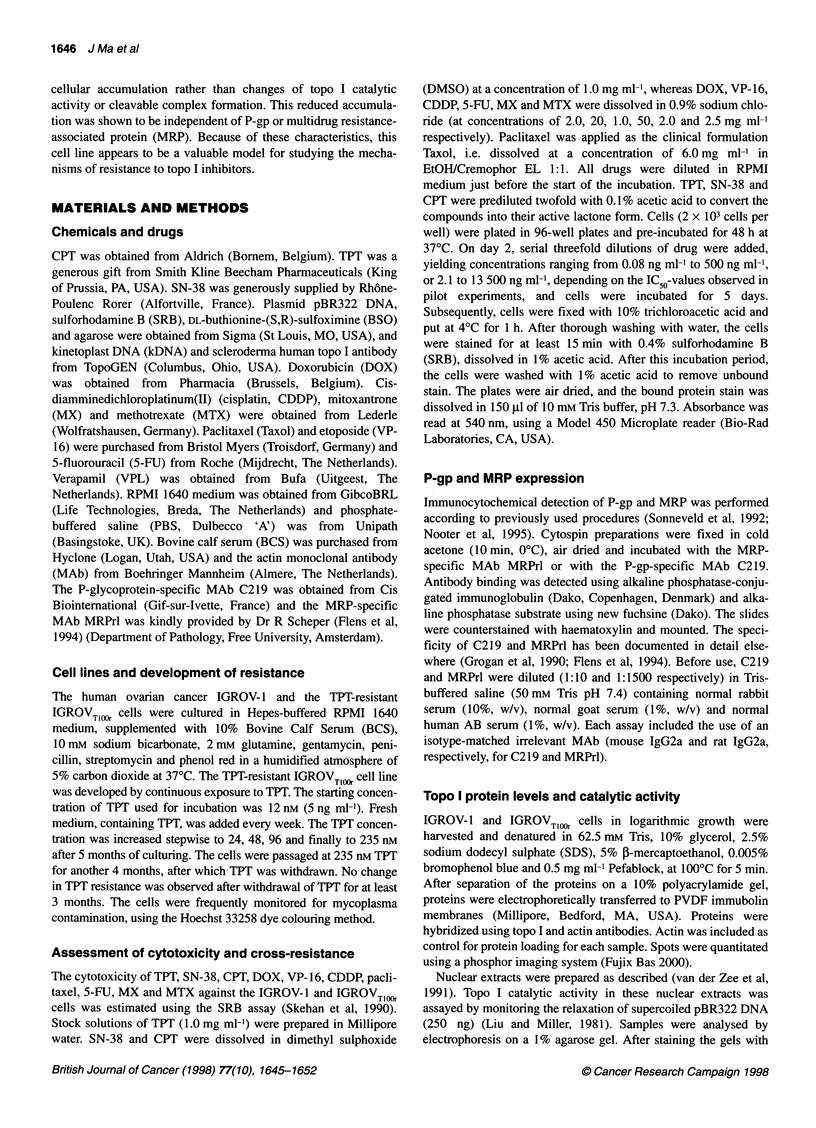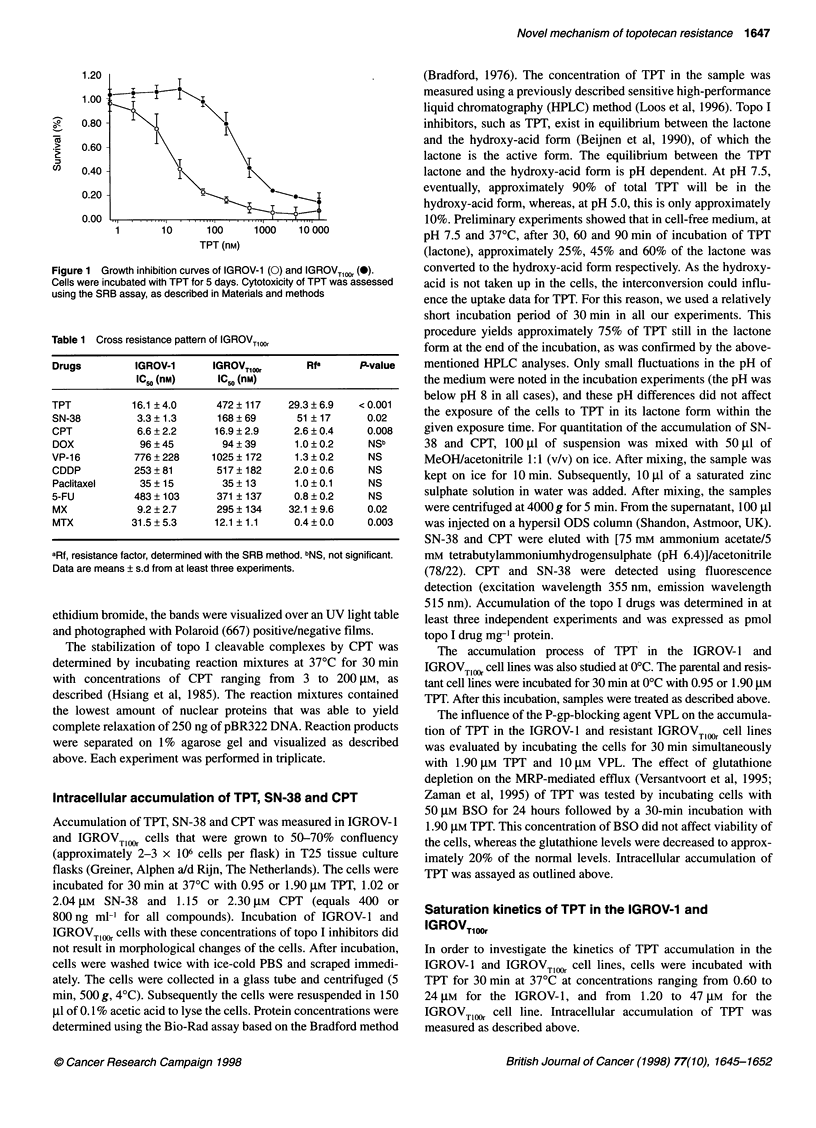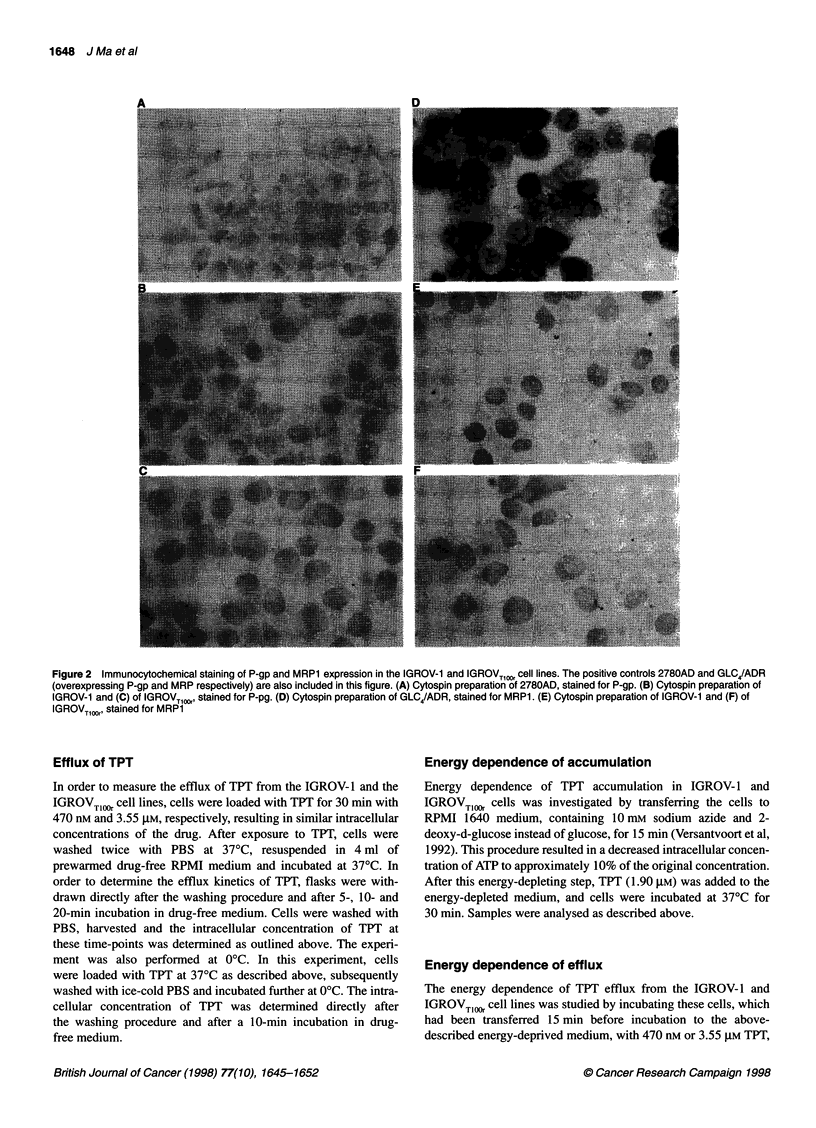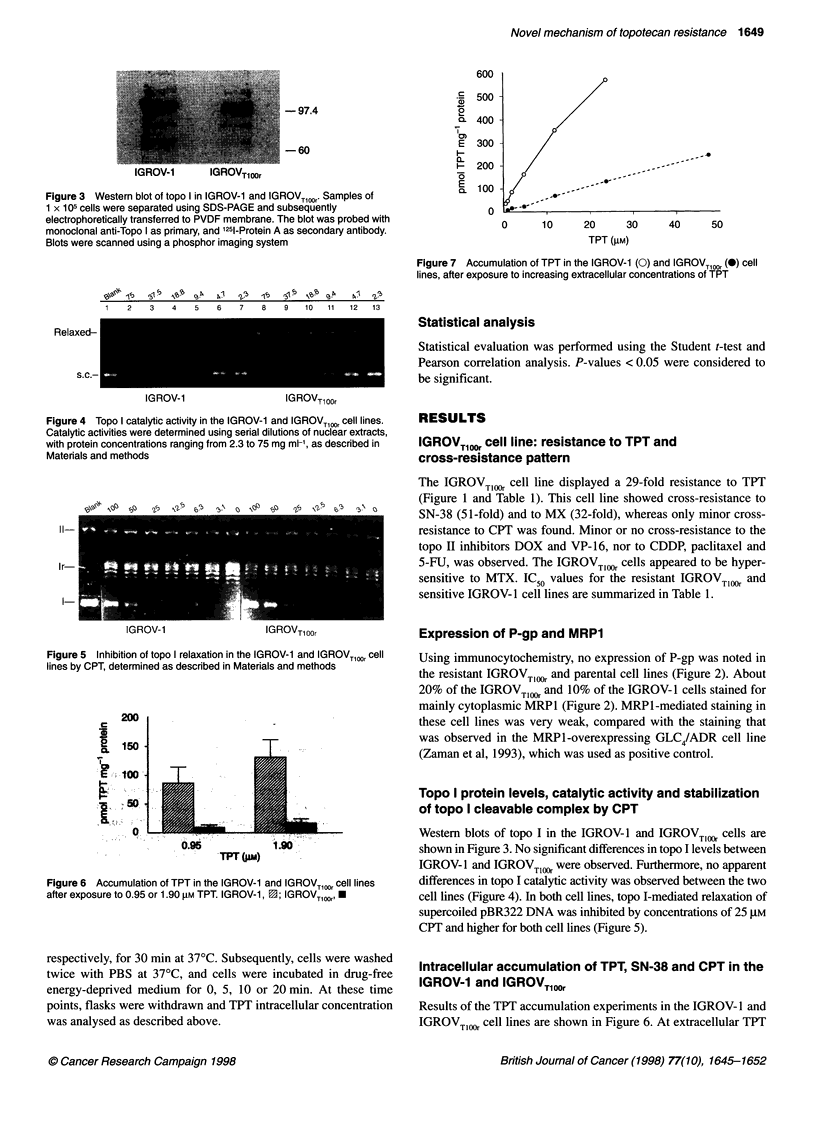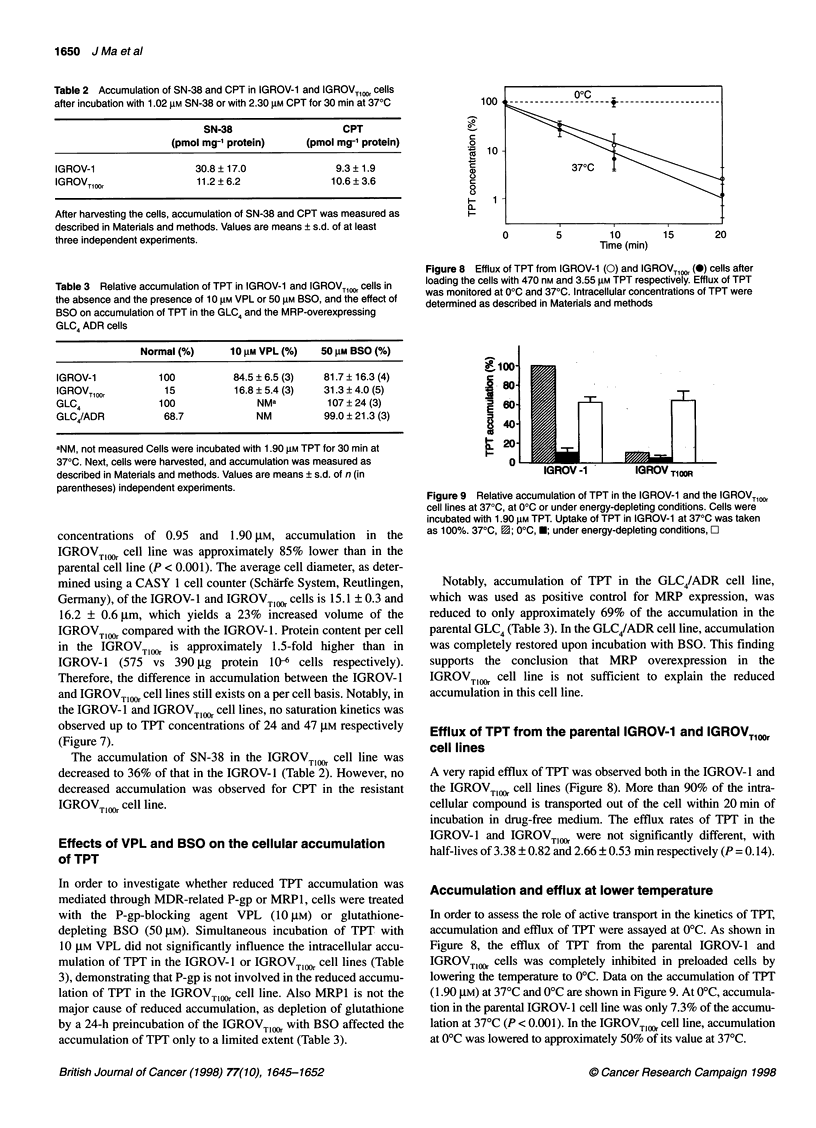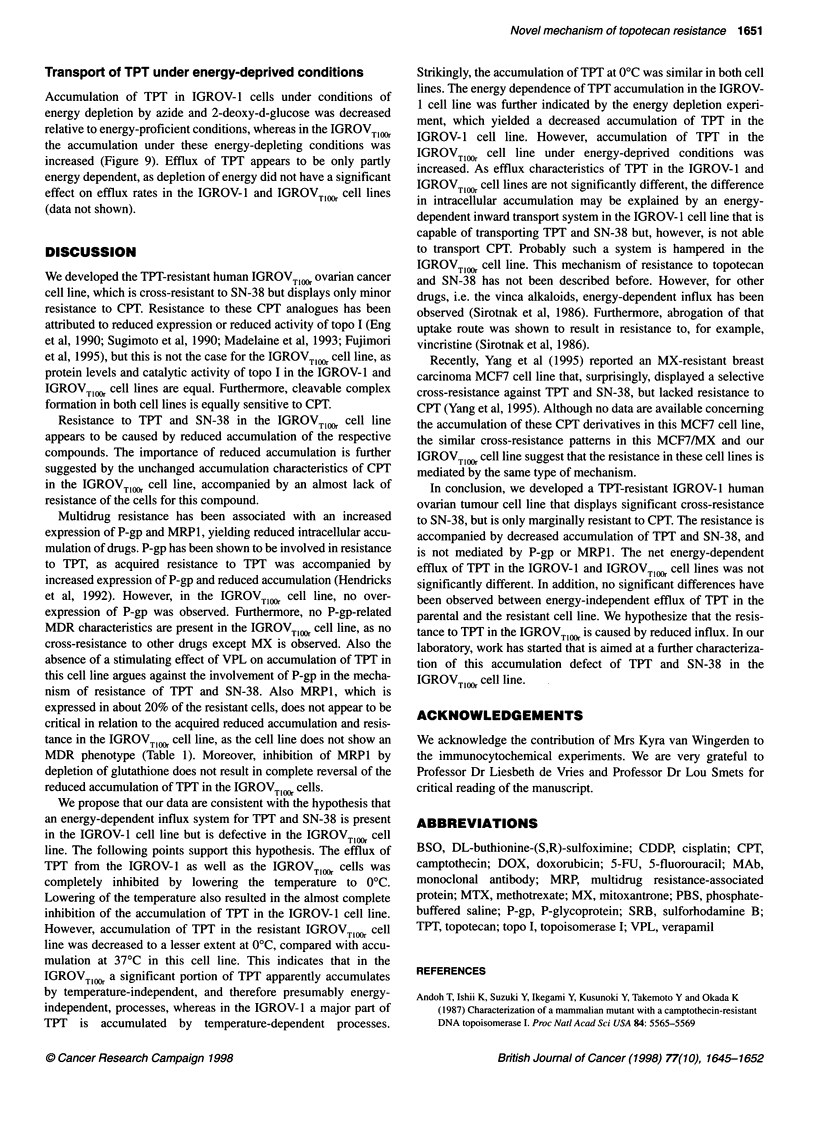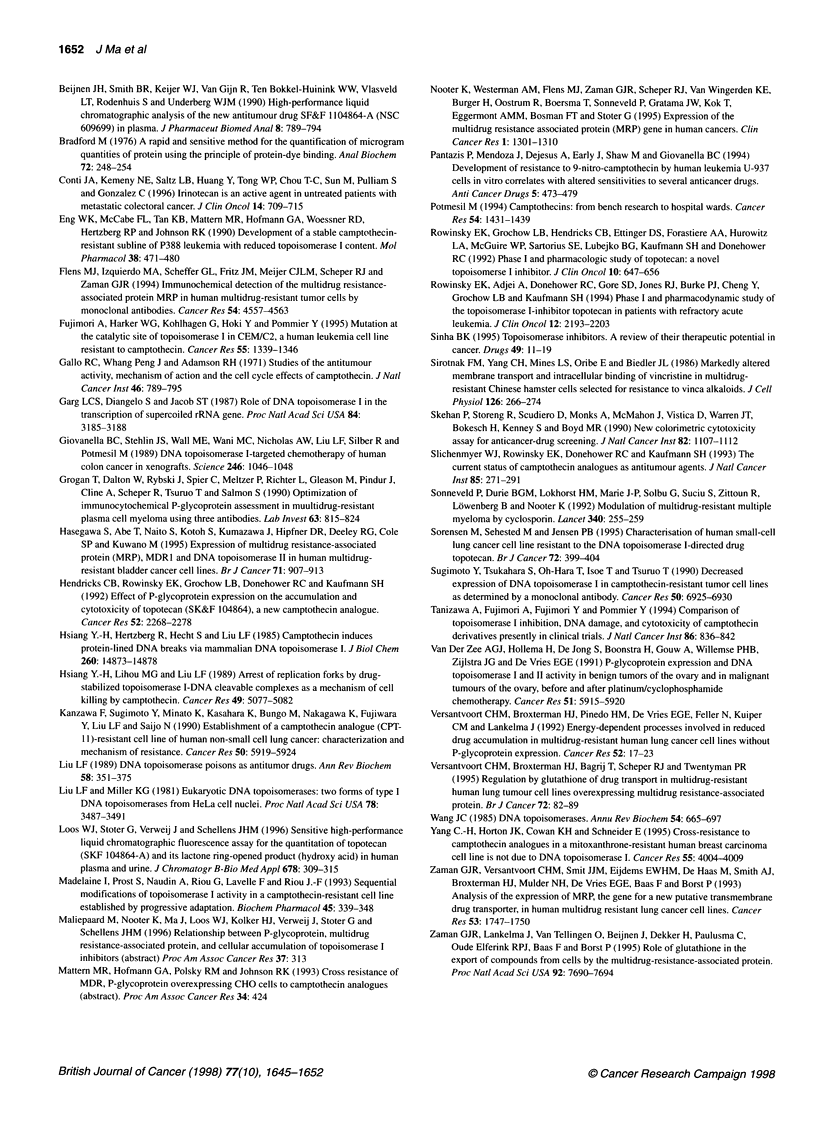# Reduced cellular accumulation of topotecan: a novel mechanism of resistance in a human ovarian cancer cell line.

**DOI:** 10.1038/bjc.1998.270

**Published:** 1998-05

**Authors:** J. Ma, M. Maliepaard, K. Nooter, W. J. Loos, H. J. Kolker, J. Verweij, G. Stoter, J. H. Schellens

**Affiliations:** Department of Medical Oncology, Rotterdam Cancer Institute (Daniel den Hoed Kliniek)/University Hospital Rotterdam, The Netherlands.

## Abstract

**Images:**


*British Journal of Cancer* (1998) **77**(10), 1645–1652
© 1998 Cancer Research Campaign

### Reduced cellular accumulation of topotecan: a novel mechanism of resistance in a human ovarian cancer cell line

**J Ma[1], M Maliepaard[2], K Nooter[1], WJ Loos[1], HJ Kolker[1], J Verweij[1], G Stoter[1] and JHM Schellens[2]**

[1]Laboratory of Experimental Chemotherapy and Pharmacology, Department of Medical Oncology, Rotterdam Cancer Institute (Daniel den Hoed Kliniek)/University Hospital Rotterdam, PO Box 5201, 3008 AE Rotterdam; [2]Department of Experimental Therapy, The Netherlands Cancer Institute, Plesmanlaan 121, 1066 CX Amsterdam, The Netherlands

**Summary** In order to unravel possible mechanisms of clinical resistance to topoisomerase I inhibitors, we developed a topotecan-resistant human IGROV-1 ovarian cancer cell line, denoted $IGROV_{T100r}$, by stepwise increased exposure to topotecan (TPT). The $IGROV_{T100r}$ cell line was 29-fold resistant to TPT and strongly cross-resistant to SN-38 (51-fold). However, the $IGROV_{T100r}$ showed only threefold resistance to camptothecin (CPT). Remarkably, this cell line was 32-fold resistant to mitoxantrone, whereas no significant cross-resistance against other cytostatic drugs was observed. No differences in topoisomerase I protein levels and catalytic activity as well as topoisomerase I cleavable complex stabilization by CPT in the IGROV-1 and $IGROV_{T100r}$ cell lines were observed, indicating that resistance in the $IGROV_{T100r}$ cell line was not related to topoisomerase I-related changes. However, resistance in the resistant $IGROV_{T100r}$ cell line to TPT and SN-38 was accompanied by decreased accumulation of the drugs to approximately 15% and 36% of that obtained in IGROV-1 respectively. No reduced accumulation was observed for CPT. Notably, accumulation of TPT in the IGROV-1 cell line decreased under energy-deprived conditions, whereas the accumulation in the $IGROV_{T100r}$ cell line increased under these energy-deprived conditions. The efflux of TPT at 37°C was very rapid in the IGROV-1 as well as the $IGROV_{T100r}$ cell line, resulting in 90% efflux within 20 min. Importantly, the efflux rates of TPT in the IGROV-1 and $IGROV_{T100r}$ cell lines were not significantly different and were shown to be independent on P-glycoprotein (P-gp) or multidrug resistance-associated protein (MRP). These results strongly suggest that the resistance of the $IGROV_{T100r}$ cell line to TPT and SN-38 is mainly caused by reduced accumulation. The reduced accumulation appears to be mediated by a novel mechanism, probably related to impaired energy-dependent uptake of these topoisomerase I drugs.

**Keywords:** topotecan; irinotecan; camptothecin; resistance; accumulation; topoisomerase I; cell line

DNA topoisomerase I (topo I) catalyses changes in DNA topology through cycles of transient DNA strand breaks and religations (Wang, 1985; Garg et al, 1987; Liu, 1989). During these events, a covalent complex, the so-called cleavable complex, is formed. Topo I-inhibiting anti-cancer agents, e.g. camptothecin (CPT) are capable of stabilizing the cleavable complex formed by topo I, and inhibit the DNA function of topo I (Gallo et al, 1971; Hsiang et al, 1985; Hsiang et al, 1989). Drug treatment results in inhibition of DNA replication and chromosomal fragmentation. Because of practical and clinical problems associated with CPT, a number of derivatives have been developed in recent years, e.g. irinotecan (CPT-11) and its active metabolite SN-38, TPT, 9-aminocamptothecin (9-AC) and GI-147211 (Sinha, 1995). These drugs show a broad spectrum of anti-cancer activity in preclinical and clinical studies (Giovanella et al, 1989; Rowinsky et al, 1992; Slichenmyer et al, 1993; Potmesil, 1994; Rowinsky et al, 1994; Tanizawa et al, 1994; Sinha, 1995; Conti et al, 1996). However, tumours may develop clinical resistance against these topo I inhibitors. For this reason, studies on the mechanisms of resistance to topo I inhibitors are warranted.

*Received 26 June 1997*
*Revised 11 November 1997*
*Accepted 17 November 1997*

*Correspondence to:* M Maliepaard

Several topo I inhibitor resistant cell lines have been established (Andoh et al, 1987; Eng et al, 1990; Kanzawa et al, 1990; Sugimoto et al, 1990; Hendricks et al, 1992; Madelaine et al, 1993; Pantazis et al, 1994; Fujimori et al, 1995; Sorensen et al, 1995). The mechanism of resistance has often been ascribed to various topo I-related changes, e.g. (1) reduced topo I catalytic activity (Madelaine et al, 1993; Fujimori et al, 1995; Sorensen et al, 1995), (2) decreased formation of cleavable complexes (Madelaine et al, 1993; Fujimori et al, 1995), (3) decreased expression of topo I (Eng et al, 1990; Sugimoto et al, 1990) and (4) a point mutation or rearrangement of topo I genes (Andoh et al, 1987; Fujimori et al, 1995). Reduced accumulation of TPT, due to P-glycoprotein (P-gp) overexpression, has been reported to add to resistance to topo I inhibitors as well (Hendricks et al, 1992; Mattern et al, 1993; Hasegawa et al, 1995). Moreover, TPT is also a substrate for the multidrug resistance-associated protein (MRP) (Maliepaard et al, 1996). Importantly, resistance to CPT does not appear to be related to P-gp or MRP (Hendricks et al, 1992; Mattern et al, 1993; Maliepaard et al, 1996). This difference between TPT and CPT may be partly caused by positive charge of TPT at physiological pH, although other factors may also be important in this respect (Mattern et al, 1993).

In this paper, we report the development and characterization of a TPT-resistant human IGROV-1 ovarian cancer cell line that is highly cross-resistant to SN-38, but displays only minor resistance to CPT. The acquisition of resistance was associated with reduced cellular accumulation rather than changes of topo I catalytic activity or cleavable complex formation. This reduced accumulation was shown to be independent of P-gp or multidrug resistance-associated protein (MRP). Because of these characteristics, this cell line appears to be a valuable model for studying the mechanisms of resistance to topo I inhibitors.

### MATERIALS AND METHODS

### Chemicals and drugs

CPT was obtained from Aldrich (Bornem, Belgium). TPT was a generous gift from Smith Kline Beecham Pharmaceuticals (King of Prussia, PA, USA). SN-38 was generously supplied by Rhône-Poulenc Rorer (Alfortville, France). Plasmid pBR322 DNA, sulforhodamine B (SRB), DL-buthionine-(S,R)-sulfoximine (BSO) and agarose were obtained from Sigma (St Louis, MO, USA), and kinetoplast DNA (kDNA) and scleroderma human topo I antibody from TopoGEN (Columbus, Ohio, USA). Doxorubicin (DOX) was obtained from Pharmacia (Brussels, Belgium). Cis-diamminedichloroplatinum(II) (cisplatin, CDDP), mitoxantrone (MX) and methotrexate (MTX) were obtained from Lederle (Wolfratshausen, Germany). Paclitaxel (Taxol) and etoposide (VP-16) were purchased from Bristol Myers (Troisdorf, Germany) and 5-fluorouracil (5-FU) from Roche (Mijdrecht, The Netherlands). Verapamil (VPL) was obtained from Bufa (Uitgeest, The Netherlands). RPMI 1640 medium was obtained from GibcoBRL (Life Technologies, Breda, The Netherlands) and phosphate-buffered saline (PBS, Dulbecco 'A') was from Unipath (Basingstoke, UK). Bovine calf serum (BCS) was purchased from Hyclone (Logan, Utah, USA) and the actin monoclonal antibody (MAb) from Boehringer Mannheim (Almere, The Netherlands). The P-glycoprotein-specific MAb C219 was obtained from Cis Biointernational (Gif-sur-Ivette, France) and the MRP-specific MAb MRPrl was kindly provided by Dr R Scheper (Flens et al, 1994) (Department of Pathology, Free University, Amsterdam).

### Cell lines and development of resistance

The human ovarian cancer IGROV-1 and the TPT-resistant $IGROV_{T100r}$ cells were cultured in Hepes-buffered RPMI 1640 medium, supplemented with 10% Bovine Calf Serum (BCS), 10 mM sodium bicarbonate, 2 mM glutamine, gentamycin, penicillin, streptomycin and phenol red in a humidified atmosphere of 5% carbon dioxide at 37°C. The TPT-resistant $IGROV_{T100r}$ cell line was developed by continuous exposure to TPT. The starting concentration of TPT used for incubation was 12 nM (5 ng $ml^{-1}$). Fresh medium, containing TPT, was added every week. The TPT concentration was increased stepwise to 24, 48, 96 and finally to 235 nM after 5 months of culturing. The cells were passaged at 235 nM TPT for another 4 months, after which TPT was withdrawn. No change in TPT resistance was observed after withdrawal of TPT for at least 3 months. The cells were frequently monitored for mycoplasma contamination, using the Hoechst 33258 dye colouring method.

### Assessment of cytotoxicity and cross-resistance

The cytotoxicity of TPT, SN-38, CPT, DOX, VP-16, CDDP, paclitaxel, 5-FU, MX and MTX against the IGROV-1 and $IGROV_{T100r}$ cells was estimated using the SRB assay (Skehan et al, 1990). Stock solutions of TPT (1.0 mg $ml^{-1}$) were prepared in Millipore water. SN-38 and CPT were dissolved in dimethyl sulphoxide (DMSO) at a concentration of 1.0 mg $ml^{-1}$, whereas DOX, VP-16, CDDP, 5-FU, MX and MTX were dissolved in 0.9% sodium chloride (at concentrations of 2.0, 20, 1.0, 50, 2.0 and 2.5 mg $ml^{-1}$ respectively). Paclitaxel was applied as the clinical formulation Taxol, i.e. dissolved at a concentration of 6.0 mg $ml^{-1}$ in EtOH/Cremophor EL 1:1. All drugs were diluted in RPMI medium just before the start of the incubation. TPT, SN-38 and CPT were prediluted twofold with 0.1% acetic acid to convert the compounds into their active lactone form. Cells ($2 \times 10^3$ cells per well) were plated in 96-well plates and pre-incubated for 48 h at 37°C. On day 2, serial threefold dilutions of drug were added, yielding concentrations ranging from 0.08 ng $ml^{-1}$ to 500 ng $ml^{-1}$, or 2.1 to 13 500 ng $ml^{-1}$, depending on the $IC_{50}$-values observed in pilot experiments, and cells were incubated for 5 days. Subsequently, cells were fixed with 10% trichloroacetic acid and put at 4°C for 1 h. After thorough washing with water, the cells were stained for at least 15 min with 0.4% sulforhodamine B (SRB), dissolved in 1% acetic acid. After this incubation period, the cells were washed with 1% acetic acid to remove unbound stain. The plates were air dried, and the bound protein stain was dissolved in 150 μl of 10 mM Tris buffer, pH 7.3. Absorbance was read at 540 nm, using a Model 450 Microplate reader (Bio-Rad Laboratories, CA, USA).

### P-gp and MRP expression

Immunocytochemical detection of P-gp and MRP was performed according to previously used procedures (Sonneveld et al, 1992; Nooter et al, 1995). Cytospin preparations were fixed in cold acetone (10 min, 0°C), air dried and incubated with the MRP-specific MAb MRPrl or with the P-gp-specific MAb C219. Antibody binding was detected using alkaline phosphatase-conjugated immunoglobulin (Dako, Copenhagen, Denmark) and alkaline phosphatase substrate using new fuchsine (Dako). The slides were counterstained with haematoxylin and mounted. The specificity of C219 and MRPrl has been documented in detail elsewhere (Grogan et al, 1990; Flens et al, 1994). Before use, C219 and MRPrl were diluted (1:10 and 1:1500 respectively) in Tris-buffered saline (50 mM Tris pH 7.4) containing normal rabbit serum (10%, w/v), normal goat serum (1%, w/v) and normal human AB serum (1%, w/v). Each assay included the use of an isotype-matched irrelevant MAb (mouse IgG2a and rat IgG2a, respectively, for C219 and MRPrl).

### Topo I protein levels and catalytic activity

IGROV-1 and $IGROV_{T100r}$ cells in logarithmic growth were harvested and denatured in 62.5 mM Tris, 10% glycerol, 2.5% sodium dodecyl sulphate (SDS), 5% β-mercaptoethanol, 0.005% bromophenol blue and 0.5 mg $ml^{-1}$ Pefablock, at 100°C for 5 min. After separation of the proteins on a 10% polyacrylamide gel, proteins were electrophoretically transferred to PVDF immubolin membranes (Millipore, Bedford, MA, USA). Proteins were hybridized using topo I and actin antibodies. Actin was included as control for protein loading for each sample. Spots were quantitated using a phosphor imaging system (Fujix Bas 2000).

Nuclear extracts were prepared as described (van der Zee et al, 1991). Topo I catalytic activity in these nuclear extracts was assayed by monitoring the relaxation of supercoiled pBR322 DNA (250 ng) (Liu and Miller, 1981). Samples were analysed by electrophoresis on a 1% agarose gel. After staining the gels with

© Cancer Research Campaign 1998

**Figure 1** Growth inhibition curves of IGROV-1 (○) and $IGROV_{T100r}$ (●). Cells were incubated with TPT for 5 days. Cytotoxicity of TPT was assessed using the SRB assay, as described in Materials and methods

**Table 1** Cross resistance pattern of $IGROV_{T100r}$

| Drugs | IGROV-1 $IC_{50}$ (nM) | $IGROV_{T100r}$ $IC_{50}$ (nM) | Rf[a] | *P*-value |
|---|---|---|---|---|
| TPT | 16.1 ± 4.0 | 472 ± 117 | 29.3 ± 6.9 | < 0.001 |
| SN-38 | 3.3 ± 1.3 | 168 ± 69 | 51 ± 17 | 0.02 |
| CPT | 6.6 ± 2.2 | 16.9 ± 2.9 | 2.6 ± 0.4 | 0.008 |
| DOX | 96 ± 45 | 94 ± 39 | 1.0 ± 0.2 | NS[b] |
| VP-16 | 776 ± 228 | 1025 ± 172 | 1.3 ± 0.2 | NS |
| CDDP | 253 ± 81 | 517 ± 182 | 2.0 ± 0.6 | NS |
| Paclitaxel | 35 ± 15 | 35 ± 13 | 1.0 ± 0.1 | NS |
| 5-FU | 483 ± 103 | 371 ± 137 | 0.8 ± 0.2 | NS |
| MX | 9.2 ± 2.7 | 295 ± 134 | 32.1 ± 9.6 | 0.02 |
| MTX | 31.5 ± 5.3 | 12.1 ± 1.1 | 0.4 ± 0.0 | 0.003 |

[a]Rf, resistance factor, determined with the SRB method. [b]NS, not significant. Data are means ± s.d from at least three experiments.

ethidium bromide, the bands were visualized over an UV light table and photographed with Polaroid (667) positive/negative films.

The stabilization of topo I cleavable complexes by CPT was determined by incubating reaction mixtures at 37°C for 30 min with concentrations of CPT ranging from 3 to 200 μM, as described (Hsiang et al, 1985). The reaction mixtures contained the lowest amount of nuclear proteins that was able to yield complete relaxation of 250 ng of pBR322 DNA. Reaction products were separated on 1% agarose gel and visualized as described above. Each experiment was performed in triplicate.

### Intracellular accumulation of TPT, SN-38 and CPT

Accumulation of TPT, SN-38 and CPT was measured in IGROV-1 and $IGROV_{T100r}$ cells that were grown to 50–70% confluency (approximately 2–3 × $10^6$ cells per flask) in T25 tissue culture flasks (Greiner, Alphen a/d Rijn, The Netherlands). The cells were incubated for 30 min at 37°C with 0.95 or 1.90 μM TPT, 1.02 or 2.04 μM SN-38 and 1.15 or 2.30 μM CPT (equals 400 or 800 ng $ml^{-1}$ for all compounds). Incubation of IGROV-1 and $IGROV_{T100r}$ cells with these concentrations of topo I inhibitors did not result in morphological changes of the cells. After incubation, cells were washed twice with ice-cold PBS and scraped immediately. The cells were collected in a glass tube and centrifuged (5 min, 500 *g*, 4°C). Subsequently the cells were resuspended in 150 μl of 0.1% acetic acid to lyse the cells. Protein concentrations were determined using the Bio-Rad assay based on the Bradford method (Bradford, 1976). The concentration of TPT in the sample was measured using a previously described sensitive high-performance liquid chromatography (HPLC) method (Loos et al, 1996). Topo I inhibitors, such as TPT, exist in equilibrium between the lactone and the hydroxy-acid form (Beijnen et al, 1990), of which the lactone is the active form. The equilibrium between the TPT lactone and the hydroxy-acid form is pH dependent. At pH 7.5, eventually, approximately 90% of total TPT will be in the hydroxy-acid form, whereas, at pH 5.0, this is only approximately 10%. Preliminary experiments showed that in cell-free medium, at pH 7.5 and 37°C, after 30, 60 and 90 min of incubation of TPT (lactone), approximately 25%, 45% and 60% of the lactone was converted to the hydroxy-acid form respectively. As the hydroxy-acid is not taken up in the cells, the interconversion could influence the uptake data for TPT. For this reason, we used a relatively short incubation period of 30 min in all our experiments. This procedure yields approximately 75% of TPT still in the lactone form at the end of the incubation, as was confirmed by the above-mentioned HPLC analyses. Only small fluctuations in the pH of the medium were noted in the incubation experiments (the pH was below pH 8 in all cases), and these pH differences did not affect the exposure of the cells to TPT in its lactone form within the given exposure time. For quantitation of the accumulation of SN-38 and CPT, 100 μl of suspension was mixed with 50 μl of MeOH/acetonitrile 1:1 (v/v) on ice. After mixing, the sample was kept on ice for 10 min. Subsequently, 10 μl of a saturated zinc sulphate solution in water was added. After mixing, the samples were centrifuged at 4000 *g* for 5 min. From the supernatant, 100 μl was injected on a hypersil ODS column (Shandon, Astmoor, UK). SN-38 and CPT were eluted with [75 mM ammonium acetate/5 mM tetrabutylammoniumhydrogensulphate (pH 6.4)]/acetonitrile (78/22). CPT and SN-38 were detected using fluorescence detection (excitation wavelength 355 nm, emission wavelength 515 nm). Accumulation of the topo I drugs was determined in at least three independent experiments and was expressed as pmol topo I drug $mg^{-1}$ protein.

The accumulation process of TPT in the IGROV-1 and $IGROV_{T100r}$ cell lines was also studied at 0°C. The parental and resistant cell lines were incubated for 30 min at 0°C with 0.95 or 1.90 μM TPT. After this incubation, samples were treated as described above.

The influence of the P-gp-blocking agent VPL on the accumulation of TPT in the IGROV-1 and resistant $IGROV_{T100r}$ cell lines was evaluated by incubating the cells for 30 min simultaneously with 1.90 μM TPT and 10 μM VPL. The effect of glutathione depletion on the MRP-mediated efflux (Versantvoort et al, 1995; Zaman et al, 1995) of TPT was tested by incubating cells with 50 μM BSO for 24 hours followed by a 30-min incubation with 1.90 μM TPT. This concentration of BSO did not affect viability of the cells, whereas the glutathione levels were decreased to approximately 20% of the normal levels. Intracellular accumulation of TPT was assayed as outlined above.

### Saturation kinetics of TPT in the IGROV-1 and $IGROV_{T100r}$

In order to investigate the kinetics of TPT accumulation in the IGROV-1 and $IGROV_{T100r}$ cell lines, cells were incubated with TPT for 30 min at 37°C at concentrations ranging from 0.60 to 24 μM for the IGROV-1, and from 1.20 to 47 μM for the $IGROV_{T100r}$ cell line. Intracellular accumulation of TPT was measured as described above.

© Cancer Research Campaign 1998 

**Figure 2** Immunocytochemical staining of P-gp and MRP1 expression in the IGROV-1 and $IGROV_{T100r}$ cell lines. The positive controls 2780AD and $GLC_4$/ADR (overexpressing P-gp and MRP respectively) are also included in this figure. (**A**) Cytospin preparation of 2780AD, stained for P-gp. (**B**) Cytospin preparation of IGROV-1 and (**C**) of $IGROV_{T100r}$, stained for P-pg. (**D**) Cytospin preparation of $GLC_4$/ADR, stained for MRP1. (**E**) Cytospin preparation of IGROV-1 and (**F**) of $IGROV_{T100r}$, stained for MRP1

### Efflux of TPT

In order to measure the efflux of TPT from the IGROV-1 and the $IGROV_{T100r}$ cell lines, cells were loaded with TPT for 30 min with 470 nM and 3.55 μM, respectively, resulting in similar intracellular concentrations of the drug. After exposure to TPT, cells were washed twice with PBS at 37°C, resuspended in 4 ml of prewarmed drug-free RPMI medium and incubated at 37°C. In order to determine the efflux kinetics of TPT, flasks were withdrawn directly after the washing procedure and after 5-, 10- and 20-min incubation in drug-free medium. Cells were washed with PBS, harvested and the intracellular concentration of TPT at these time-points was determined as outlined above. The experiment was also performed at 0°C. In this experiment, cells were loaded with TPT at 37°C as described above, subsequently washed with ice-cold PBS and incubated further at 0°C. The intracellular concentration of TPT was determined directly after the washing procedure and after a 10-min incubation in drug-free medium.

### Energy dependence of accumulation

Energy dependence of TPT accumulation in IGROV-1 and $IGROV_{T100r}$ cells was investigated by transferring the cells to RPMI 1640 medium, containing 10 mM sodium azide and 2-deoxy-d-glucose instead of glucose, for 15 min (Versantvoort et al, 1992). This procedure resulted in a decreased intracellular concentration of ATP to approximately 10% of the original concentration. After this energy-depleting step, TPT (1.90 μM) was added to the energy-depleted medium, and cells were incubated at 37°C for 30 min. Samples were analysed as described above.

### Energy dependence of efflux

The energy dependence of TPT efflux from the IGROV-1 and $IGROV_{T100r}$ cell lines was studied by incubating these cells, which had been transferred 15 min before incubation to the above-described energy-deprived medium, with 470 nM or 3.55 μM TPT,

© Cancer Research Campaign 1998

**Figure 3** Western blot of topo I in IGROV-1 and IGROV$_{T100r}$. Samples of $1 \times 10^5$ cells were separated using SDS-PAGE and subsequently electrophoretically transferred to PVDF membrane. The blot was probed with monoclonal anti-Topo I as primary, and $^{125}$I-Protein A as secondary antibody. Blots were scanned using a phosphor imaging system

**Figure 4** Topo I catalytic activity in the IGROV-1 and IGROV$_{T100r}$ cell lines. Catalytic activities were determined using serial dilutions of nuclear extracts, with protein concentrations ranging from 2.3 to 75 mg ml$^{-1}$, as described in Materials and methods

**Figure 5** Inhibition of topo I relaxation in the IGROV-1 and IGROV$_{T100r}$ cell lines by CPT, determined as described in Materials and methods

**Figure 6** Accumulation of TPT in the IGROV-1 and IGROV$_{T100r}$ cell lines after exposure to 0.95 or 1.90 μM TPT. IGROV-1, ▨; IGROV$_{T100r}$, ■

respectively, for 30 min at 37°C. Subsequently, cells were washed twice with PBS at 37°C, and cells were incubated in drug-free energy-deprived medium for 0, 5, 10 or 20 min. At these time points, flasks were withdrawn and TPT intracellular concentration was analysed as described above.

**Figure 7** Accumulation of TPT in the IGROV-1 (○) and IGROV$_{T100r}$ (●) cell lines, after exposure to increasing extracellular concentrations of TPT

### Statistical analysis

Statistical evaluation was performed using the Student *t*-test and Pearson correlation analysis. *P*-values < 0.05 were considered to be significant.

### RESULTS

### IGROV$_{T100r}$ cell line: resistance to TPT and cross-resistance pattern

The IGROV$_{T100r}$ cell line displayed a 29-fold resistance to TPT (Figure 1 and Table 1). This cell line showed cross-resistance to SN-38 (51-fold) and to MX (32-fold), whereas only minor cross-resistance to CPT was found. Minor or no cross-resistance to the topo II inhibitors DOX and VP-16, nor to CDDP, paclitaxel and 5-FU, was observed. The IGROV$_{T100r}$ cells appeared to be hypersensitive to MTX. $IC_{50}$ values for the resistant IGROV$_{T100r}$ and sensitive IGROV-1 cell lines are summarized in Table 1.

### Expression of P-gp and MRP1

Using immunocytochemistry, no expression of P-gp was noted in the resistant IGROV$_{T100r}$ and parental cell lines (Figure 2). About 20% of the IGROV$_{T100r}$ and 10% of the IGROV-1 cells stained for mainly cytoplasmic MRP1 (Figure 2). MRP1-mediated staining in these cell lines was very weak, compared with the staining that was observed in the MRP1-overexpressing GLC$_4$/ADR cell line (Zaman et al, 1993), which was used as positive control.

### Topo I protein levels, catalytic activity and stabilization of topo I cleavable complex by CPT

Western blots of topo I in the IGROV-1 and IGROV$_{T100r}$ cells are shown in Figure 3. No significant differences in topo I levels between IGROV-1 and IGROV$_{T100r}$ were observed. Furthermore, no apparent differences in topo I catalytic activity was observed between the two cell lines (Figure 4). In both cell lines, topo I-mediated relaxation of supercoiled pBR322 DNA was inhibited by concentrations of 25 μM CPT and higher for both cell lines (Figure 5).

### Intracellular accumulation of TPT, SN-38 and CPT in the IGROV-1 and IGROV$_{T100r}$

Results of the TPT accumulation experiments in the IGROV-1 and IGROV$_{T100r}$ cell lines are shown in Figure 6. At extracellular TPT

© Cancer Research Campaign 1998 

**Table 2** Accumulation of SN-38 and CPT in IGROV-1 and $IGROV_{T100r}$ cells after incubation with 1.02 μM SN-38 or with 2.30 μM CPT for 30 min at 37°C

| | SN-38 (pmol mg⁻¹ protein) | CPT (pmol mg⁻¹ protein) |
|---|---|---|
| IGROV-1 | 30.8 ± 17.0 | 9.3 ± 1.9 |
| $IGROV_{T100r}$ | 11.2 ± 6.2 | 10.6 ± 3.6 |

After harvesting the cells, accumulation of SN-38 and CPT was measured as described in Materials and methods. Values are means ± s.d. of at least three independent experiments.

**Table 3** Relative accumulation of TPT in IGROV-1 and $IGROV_{T100r}$ cells in the absence and the presence of 10 μM VPL or 50 μM BSO, and the effect of BSO on accumulation of TPT in the $GLC_4$ and the MRP-overexpressing $GLC_4$ ADR cells

| | Normal (%) | 10 μM VPL (%) | 50 μM BSO (%) |
|---|---|---|---|
| IGROV-1 | 100 | 84.5 ± 6.5 (3) | 81.7 ± 16.3 (4) |
| $IGROV_{T100r}$ | 15 | 16.8 ± 5.4 (3) | 31.3 ± 4.0 (5) |
| $GLC_4$ | 100 | NM[a] | 107 ± 24 (3) |
| $GLC_4$/ADR | 68.7 | NM | 99.0 ± 21.3 (3) |

[a]NM, not measured Cells were incubated with 1.90 μM TPT for 30 min at 37°C. Next, cells were harvested, and accumulation was measured as described in Materials and methods. Values are means ± s.d. of *n* (in parentheses) independent experiments.

concentrations of 0.95 and 1.90 μM, accumulation in the $IGROV_{T100r}$ cell line was approximately 85% lower than in the parental cell line ($P < 0.001$). The average cell diameter, as determined using a CASY 1 cell counter (Schärfe System, Reutlingen, Germany), of the IGROV-1 and $IGROV_{T100r}$ cells is 15.1 ± 0.3 and 16.2 ± 0.6 μm, which yields a 23% increased volume of the $IGROV_{T100r}$ compared with the IGROV-1. Protein content per cell in the $IGROV_{T100r}$ is approximately 1.5-fold higher than in IGROV-1 (575 vs 390 μg protein $10^{-6}$ cells respectively). Therefore, the difference in accumulation between the IGROV-1 and $IGROV_{T100r}$ cell lines still exists on a per cell basis. Notably, in the IGROV-1 and $IGROV_{T100r}$ cell lines, no saturation kinetics was observed up to TPT concentrations of 24 and 47 μM respectively (Figure 7).

The accumulation of SN-38 in the $IGROV_{T100r}$ cell line was decreased to 36% of that in the IGROV-1 (Table 2). However, no decreased accumulation was observed for CPT in the resistant $IGROV_{T100r}$ cell line.

### Effects of VPL and BSO on the cellular accumulation of TPT

In order to investigate whether reduced TPT accumulation was mediated through MDR-related P-gp or MRP1, cells were treated with the P-gp-blocking agent VPL (10 μM) or glutathione-depleting BSO (50 μM). Simultaneous incubation of TPT with 10 μM VPL did not significantly influence the intracellular accumulation of TPT in the IGROV-1 or $IGROV_{T100r}$ cell lines (Table 3), demonstrating that P-gp is not involved in the reduced accumulation of TPT in the $IGROV_{T100r}$ cell line. Also MRP1 is not the major cause of reduced accumulation, as depletion of glutathione by a 24-h preincubation of the $IGROV_{T100r}$ with BSO affected the accumulation of TPT only to a limited extent (Table 3).

**Figure 8** Efflux of TPT from IGROV-1 (○) and $IGROV_{T100r}$ (●) cells after loading the cells with 470 nM and 3.55 μM TPT respectively. Efflux of TPT was monitored at 0°C and 37°C. Intracellular concentrations of TPT were determined as described in Materials and methods

**Figure 9** Relative accumulation of TPT in the IGROV-1 and the $IGROV_{T100r}$ cell lines at 37°C, at 0°C or under energy-depleting conditions. Cells were incubated with 1.90 μM TPT. Uptake of TPT in IGROV-1 at 37°C was taken as 100%. 37°C, ▨; 0°C, ■; under energy-depleting conditions, □

Notably, accumulation of TPT in the $GLC_4$/ADR cell line, which was used as positive control for MRP expression, was reduced to only approximately 69% of the accumulation in the parental $GLC_4$ (Table 3). In the $GLC_4$/ADR cell line, accumulation was completely restored upon incubation with BSO. This finding supports the conclusion that MRP overexpression in the $IGROV_{T100r}$ cell line is not sufficient to explain the reduced accumulation in this cell line.

### Efflux of TPT from the parental IGROV-1 and $IGROV_{T100r}$ cell lines

A very rapid efflux of TPT was observed both in the IGROV-1 and the $IGROV_{T100r}$ cell lines (Figure 8). More than 90% of the intracellular compound is transported out of the cell within 20 min of incubation in drug-free medium. The efflux rates of TPT in the IGROV-1 and $IGROV_{T100r}$ were not significantly different, with half-lives of 3.38 ± 0.82 and 2.66 ± 0.53 min respectively ($P = 0.14$).

### Accumulation and efflux at lower temperature

In order to assess the role of active transport in the kinetics of TPT, accumulation and efflux of TPT were assayed at 0°C. As shown in Figure 8, the efflux of TPT from the parental IGROV-1 and $IGROV_{T100r}$ cells was completely inhibited in preloaded cells by lowering the temperature to 0°C. Data on the accumulation of TPT (1.90 μM) at 37°C and 0°C are shown in Figure 9. At 0°C, accumulation in the parental IGROV-1 cell line was only 7.3% of the accumulation at 37°C ($P < 0.001$). In the $IGROV_{T100r}$ cell line, accumulation at 0°C was lowered to approximately 50% of its value at 37°C.

© Cancer Research Campaign 1998

### Transport of TPT under energy-deprived conditions

Accumulation of TPT in IGROV-1 cells under conditions of energy depletion by azide and 2-deoxy-d-glucose was decreased relative to energy-proficient conditions, whereas in the $IGROV_{T100r}$ the accumulation under these energy-depleting conditions was increased (Figure 9). Efflux of TPT appears to be only partly energy dependent, as depletion of energy did not have a significant effect on efflux rates in the IGROV-1 and $IGROV_{T100r}$ cell lines (data not shown).

### DISCUSSION

We developed the TPT-resistant human $IGROV_{T100r}$ ovarian cancer cell line, which is cross-resistant to SN-38 but displays only minor resistance to CPT. Resistance to these CPT analogues has been attributed to reduced expression or reduced activity of topo I (Eng et al, 1990; Sugimoto et al, 1990; Madelaine et al, 1993; Fujimori et al, 1995), but this is not the case for the $IGROV_{T100r}$ cell line, as protein levels and catalytic activity of topo I in the IGROV-1 and $IGROV_{T100r}$ cell lines are equal. Furthermore, cleavable complex formation in both cell lines is equally sensitive to CPT.

Resistance to TPT and SN-38 in the $IGROV_{T100r}$ cell line appears to be caused by reduced accumulation of the respective compounds. The importance of reduced accumulation is further suggested by the unchanged accumulation characteristics of CPT in the $IGROV_{T100r}$ cell line, accompanied by an almost lack of resistance of the cells for this compound.

Multidrug resistance has been associated with an increased expression of P-gp and MRP1, yielding reduced intracellular accumulation of drugs. P-gp has been shown to be involved in resistance to TPT, as acquired resistance to TPT was accompanied by increased expression of P-gp and reduced accumulation (Hendricks et al, 1992). However, in the $IGROV_{T100r}$ cell line, no overexpression of P-gp was observed. Furthermore, no P-gp-related MDR characteristics are present in the $IGROV_{T100r}$ cell line, as no cross-resistance to other drugs except MX is observed. Also the absence of a stimulating effect of VPL on accumulation of TPT in this cell line argues against the involvement of P-gp in the mechanism of resistance of TPT and SN-38. Also MRP1, which is expressed in about 20% of the resistant cells, does not appear to be critical in relation to the acquired reduced accumulation and resistance in the $IGROV_{T100r}$ cell line, as the cell line does not show an MDR phenotype (Table 1). Moreover, inhibition of MRP1 by depletion of glutathione does not result in complete reversal of the reduced accumulation of TPT in the $IGROV_{T100r}$ cells.

We propose that our data are consistent with the hypothesis that an energy-dependent influx system for TPT and SN-38 is present in the IGROV-1 cell line but is defective in the $IGROV_{T100r}$ cell line. The following points support this hypothesis. The efflux of TPT from the IGROV-1 as well as the $IGROV_{T100r}$ cells was completely inhibited by lowering the temperature to 0°C. Lowering of the temperature also resulted in the almost complete inhibition of the accumulation of TPT in the IGROV-1 cell line. However, accumulation of TPT in the resistant $IGROV_{T100r}$ cell line was decreased to a lesser extent at 0°C, compared with accumulation at 37°C in this cell line. This indicates that in the $IGROV_{T100r}$ a significant portion of TPT apparently accumulates by temperature-independent, and therefore presumably energy-independent, processes, whereas in the IGROV-1 a major part of TPT is accumulated by temperature-dependent processes. Strikingly, the accumulation of TPT at 0°C was similar in both cell lines. The energy dependence of TPT accumulation in the IGROV-1 cell line was further indicated by the energy depletion experiment, which yielded a decreased accumulation of TPT in the IGROV-1 cell line. However, accumulation of TPT in the $IGROV_{T100r}$ cell line under energy-deprived conditions was increased. As efflux characteristics of TPT in the IGROV-1 and $IGROV_{T100r}$ cell lines are not significantly different, the difference in intracellular accumulation may be explained by an energy-dependent inward transport system in the IGROV-1 cell line that is capable of transporting TPT and SN-38 but, however, is not able to transport CPT. Probably such a system is hampered in the $IGROV_{T100r}$ cell line. This mechanism of resistance to topotecan and SN-38 has not been described before. However, for other drugs, i.e. the vinca alkaloids, energy-dependent influx has been observed (Sirotnak et al, 1986). Furthermore, abrogation of that uptake route was shown to result in resistance to, for example, vincristine (Sirotnak et al, 1986).

Recently, Yang et al (1995) reported an MX-resistant breast carcinoma MCF7 cell line that, surprisingly, displayed a selective cross-resistance against TPT and SN-38, but lacked resistance to CPT (Yang et al, 1995). Although no data are available concerning the accumulation of these CPT derivatives in this MCF7 cell line, the similar cross-resistance patterns in this MCF7/MX and our $IGROV_{T100r}$ cell line suggest that the resistance in these cell lines is mediated by the same type of mechanism.

In conclusion, we developed a TPT-resistant IGROV-1 human ovarian tumour cell line that displays significant cross-resistance to SN-38, but is only marginally resistant to CPT. The resistance is accompanied by decreased accumulation of TPT and SN-38, and is not mediated by P-gp or MRP1. The net energy-dependent efflux of TPT in the IGROV-1 and $IGROV_{T100r}$ cell lines was not significantly different. In addition, no significant differences have been observed between energy-independent efflux of TPT in the parental and the resistant cell line. We hypothesize that the resistance to TPT in the $IGROV_{T100r}$ is caused by reduced influx. In our laboratory, work has started that is aimed at a further characterization of this accumulation defect of TPT and SN-38 in the $IGROV_{T100r}$ cell line.

### ACKNOWLEDGEMENTS

We acknowledge the contribution of Mrs Kyra van Wingerden to the immunocytochemical experiments. We are very grateful to Professor Dr Liesbeth de Vries and Professor Dr Lou Smets for critical reading of the manuscript.

### ABBREVIATIONS

BSO, DL-buthionine-(S,R)-sulfoximine; CDDP, cisplatin; CPT, camptothecin; DOX, doxorubicin; 5-FU, 5-fluorouracil; MAb, monoclonal antibody; MRP, multidrug resistance-associated protein; MTX, methotrexate; MX, mitoxantrone; PBS, phosphate-buffered saline; P-gp, P-glycoprotein; SRB, sulforhodamine B; TPT, topotecan; topo I, topoisomerase I; VPL, verapamil

#### REFERENCES

Andoh T, Ishii K, Suzuki Y, Ikegami Y, Kusunoki Y, Takemoto Y and Okada K (1987) Characterization of a mammalian mutant with a camptothecin-resistant DNA topoisomerase I. *Proc Natl Acad Sci USA* **84**: 5565–5569

© Cancer Research Campaign 1998

Beijnen JH, Smith BR, Keijer WJ, Van Gijn R, Ten Bokkel-Huinink WW, Vlasveld LT, Rodenhuis S and Underberg WJM (1990) High-performance liquid chromatographic analysis of the new antitumour drug SF&F 1104864-A (NSC 609699) in plasma. *J Pharmaceut Biomed Anal* **8**: 789–794

Bradford M (1976) A rapid and sensitive method for the quantification of microgram quantities of protein using the principle of protein-dye binding. *Anal Biochem* **72**: 248–254

Conti JA, Kemeny NE, Saltz LB, Huang Y, Tong WP, Chou T-C, Sun M, Pulliam S and Gonzalez C (1996) Irinotecan is an active agent in untreated patients with metastatic colectoral cancer. *J Clin Oncol* **14**: 709–715

Eng WK, McCabe FL, Tan KB, Mattern MR, Hofmann GA, Woessner RD, Hertzberg RP and Johnson RK (1990) Development of a stable camptothecin-resistant subline of P388 leukemia with reduced topoisomerase I content. *Mol Pharmacol* **38**: 471–480

Flens MJ, Izquierdo MA, Scheffer GL, Fritz JM, Meijer CJLM, Scheper RJ and Zaman GJR (1994) Immunochemical detection of the multidrug resistance-associated protein MRP in human multidrug-resistant tumor cells by monoclonal antibodies. *Cancer Res* **54**: 4557–4563

Fujimori A, Harker WG, Kohlhagen G, Hoki Y and Pommier Y (1995) Mutation at the catalytic site of topoisomerase I in CEM/C2, a human leukemia cell line resistant to camptothecin. *Cancer Res* **55**: 1339–1346

Gallo RC, Whang Peng J and Adamson RH (1971) Studies of the antitumour activity, mechanism of action and the cell cycle effects of camptothecin. *J Natl Cancer Inst* **46**: 789–795

Garg LCS, Diangelo S and Jacob ST (1987) Role of DNA topoisomerase I in the transcription of supercoiled rRNA gene. *Proc Natl Acad Sci USA* **84**: 3185–3188

Giovanella BC, Stehlin JS, Wall ME, Wani MC, Nicholas AW, Liu LF, Silber R and Potmesil M (1989) DNA topoisomerase I-targeted chemotherapy of human colon cancer in xenografts. *Science* **246**: 1046–1048

Grogan T, Dalton W, Rybski J, Spier C, Meltzer P, Richter L, Gleason M, Pindur J, Cline A, Scheper R, Tsuruo T and Salmon S (1990) Optimization of immunocytochemical P-glycoprotein assessment in muultidrug-resistant plasma cell myeloma using three antibodies. *Lab Invest* **63**: 815–824

Hasegawa S, Abe T, Naito S, Kotoh S, Kumazawa J, Hipfner DR, Deeley RG, Cole SP and Kuwano M (1995) Expression of multidrug resistance-associated protein (MRP), MDR1 and DNA topoisomerase II in human multidrug-resistant bladder cancer cell lines. *Br J Cancer* **71**: 907–913

Hendricks CB, Rowinsky EK, Grochow LB, Donehower RC and Kaufmann SH (1992) Effect of P-glycoprotein expression on the accumulation and cytotoxicity of topotecan (SK&F 104864), a new camptothecin analogue. *Cancer Res* **52**: 2268–2278

Hsiang Y.-H, Hertzberg R, Hecht S and Liu LF (1985) Camptothecin induces protein-lined DNA breaks via mammalian DNA topoisomerase I. *J Biol Chem* **260**: 14873–14878

Hsiang Y.-H, Lihou MG and Liu LF (1989) Arrest of replication forks by drug-stabilized topoisomerase I-DNA cleavable complexes as a mechanism of cell killing by camptothecin. *Cancer Res* **49**: 5077–5082

Kanzawa F, Sugimoto Y, Minato K, Kasahara K, Bungo M, Nakagawa K, Fujiwara Y, Liu LF and Saijo N (1990) Establishment of a camptothecin analogue (CPT-11)-resistant cell line of human non-small cell lung cancer: characterization and mechanism of resistance. *Cancer Res* **50**: 5919–5924

Liu LF (1989) DNA topoisomerase poisons as antitumor drugs. *Ann Rev Biochem* **58**: 351–375

Liu LF and Miller KG (1981) Eukaryotic DNA topoisomerases: two forms of type I DNA topoisomerases from HeLa cell nuclei. *Proc Natl Acad Sci USA* **78**: 3487–3491

Loos WJ, Stoter G, Verweij J and Schellens JHM (1996) Sensitive high-performance liquid chromatographic fluorescence assay for the quantitation of topotecan (SKF 104864-A) and its lactone ring-opened product (hydroxy acid) in human plasma and urine. *J Chromatogr B-Bio Med Appl* **678**: 309–315

Madelaine I, Prost S, Naudin A, Riou G, Lavelle F and Riou J.-F (1993) Sequential modifications of topoisomerase I activity in a camptothecin-resistant cell line established by progressive adaptation. *Biochem Pharmacol* **45**: 339–348

Maliepaard M, Nooter K, Ma J, Loos WJ, Kolker HJ, Verweij J, Stoter G and Schellens JHM (1996) Relationship between P-glycoprotein, multidrug resistance-associated protein, and cellular accumulation of topoisomerase I inhibitors (abstract) *Proc Am Assoc Cancer Res* **37**: 313

Mattern MR, Hofmann GA, Polsky RM and Johnson RK (1993) Cross resistance of MDR, P-glycoprotein overexpressing CHO cells to camptothecin analogues (abstract). *Proc Am Assoc Cancer Res* **34**: 424

Nooter K, Westerman AM, Flens MJ, Zaman GJR, Scheper RJ, Van Wingerden KE, Burger H, Oostrum R, Boersma T, Sonneveld P, Gratama JW, Kok T, Eggermont AMM, Bosman FT and Stoter G (1995) Expression of the multidrug resistance associated protein (MRP) gene in human cancers. *Clin Cancer Res* **1**: 1301–1310

Pantazis P, Mendoza J, Dejesus A, Early J, Shaw M and Giovanella BC (1994) Development of resistance to 9-nitro-camptothecin by human leukemia U-937 cells in vitro correlates with altered sensitivities to several anticancer drugs. *Anti Cancer Drugs* **5**: 473–479

Potmesil M (1994) Camptothecins: from bench research to hospital wards. *Cancer Res* **54**: 1431–1439

Rowinsky EK, Grochow LB, Hendricks CB, Ettinger DS, Forastiere AA, Hurowitz LA, McGuire WP, Sartorius SE, Lubejko BG, Kaufmann SH and Donehower RC (1992) Phase I and pharmacologic study of topotecan: a novel topoisomerse I inhibitor. *J Clin Oncol* **10**: 647–656

Rowinsky EK, Adjei A, Donehower RC, Gore SD, Jones RJ, Burke PJ, Cheng Y, Grochow LB and Kaufmann SH (1994) Phase I and pharmacodynamic study of the topoisomerase I-inhibitor topotecan in patients with refractory acute leukemia. *J Clin Oncol* **12**: 2193–2203

Sinha BK (1995) Topoisomerase inhibitors. A review of their therapeutic potential in cancer. *Drugs* **49**: 11–19

Sirotnak FM, Yang CH, Mines LS, Oribe E and Biedler JL (1986) Markedly altered membrane transport and intracellular binding of vincristine in multidrug-resistant Chinese hamster cells selected for resistance to vinca alkaloids. *J Cell Physiol* **126**: 266–274

Skehan P, Storeng R, Scudiero D, Monks A, McMahon J, Vistica D, Warren JT, Bokesch H, Kenney S and Boyd MR (1990) New colorimetric cytotoxicity assay for anticancer-drug screening. *J Natl Cancer Inst* **82**: 1107–1112

Slichenmyer WJ, Rowinsky EK, Donehower RC and Kaufmann SH (1993) The current status of camptothecin analogues as antitumour agents. *J Natl Cancer Inst* **85**: 271–291

Sonneveld P, Durie BGM, Lokhorst HM, Marie J-P, Solbu G, Suciu S, Zittoun R, Löwenberg B and Nooter K (1992) Modulation of multidrug-resistant multiple myeloma by cyclosporin. *Lancet* **340**: 255–259

Sorensen M, Sehested M and Jensen PB (1995) Characterisation of human small-cell lung cancer cell line resistant to the DNA topoisomerase I-directed drug topotecan. *Br J Cancer* **72**: 399–404

Sugimoto Y, Tsukahara S, Oh-Hara T, Isoe T and Tsuruo T (1990) Decreased expression of DNA topoisomerase I in camptothecin-resistant tumor cell lines as determined by a monoclonal antibody. *Cancer Res* **50**: 6925–6930

Tanizawa A, Fujimori A, Fujimori Y and Pommier Y (1994) Comparison of topoisomerase I inhibition, DNA damage, and cytotoxicity of camptothecin derivatives presently in clinical trials. *J Natl Cancer Inst* **86**: 836–842

Van Der Zee AGJ, Hollema H, De Jong S, Boonstra H, Gouw A, Willemse PHB, Zijlstra JG and De Vries EGE (1991) P-glycoprotein expression and DNA topoisomerase I and II activity in benign tumors of the ovary and in malignant tumours of the ovary, before and after platinum/cyclophosphamide chemotherapy. *Cancer Res* **51**: 5915–5920

Versantvoort CHM, Broxterman HJ, Pinedo HM, De Vries EGE, Feller N, Kuiper CM and Lankelma J (1992) Energy-dependent processes involved in reduced drug accumulation in multidrug-resistant human lung cancer cell lines without P-glycoprotein expression. *Cancer Res* **52**: 17–23

Versantvoort CHM, Broxterman HJ, Bagrij T, Scheper RJ and Twentyman PR (1995) Regulation by glutathione of drug transport in multidrug-resistant human lung tumour cell lines overexpressing multidrug resistance-associated protein. *Br J Cancer* **72**: 82–89

Wang JC (1985) DNA topoisomerases. *Annu Rev Biochem* **54**: 665–697

Yang C.-H, Horton JK, Cowan KH and Schneider E (1995) Cross-resistance to camptothecin analogues in a mitoxanthrone-resistant human breast carcinoma cell line is not due to DNA topoisomerase I. *Cancer Res* **55**: 4004–4009

Zaman GJR, Versantvoort CHM, Smit JJM, Eijdems EWHM, De Haas M, Smith AJ, Broxterman HJ, Mulder NH, De Vries EGE, Baas F and Borst P (1993) Analysis of the expression of MRP, the gene for a new putative transmembrane drug transporter, in human multidrug resistant lung cancer cell lines. *Cancer Res* **53**: 1747–1750

Zaman GJR, Lankelma J, Van Tellingen O, Beijnen J, Dekker H, Paulusma C, Oude Elferink RPJ, Baas F and Borst P (1995) Role of glutathione in the export of compounds from cells by the multidrug-resistance-associated protein. *Proc Natl Acad Sci USA* **92**: 7690–7694

© Cancer Research Campaign 1998